# Mouse Genetic Background Affects Transfer of an Antibiotic Resistance Plasmid in the Gastrointestinal Tract

**DOI:** 10.1128/mSphere.00847-19

**Published:** 2020-01-29

**Authors:** Logan C. Ott, Zachary R. Stromberg, Graham A. J. Redweik, Michael J. Wannemuehler, Melha Mellata

**Affiliations:** a Department of Food Science and Human Nutrition, Iowa State University, Ames, Iowa, USA; b Department of Veterinary Microbiology and Preventive Medicine, Iowa State University, Ames, Iowa, USA; University of Michigan—Ann Arbor

**Keywords:** horizontal gene transfer, gut microbiota, plasmid, antibiotic resistance, altered Schaedler flora

## Abstract

Dissemination of antibiotic resistance (AR) genes, often on plasmids, leads to antibiotic-resistant bacterial infections, which is a major problem for animal and public health. Bacterial conjugation is the primary route of AR gene transfer in the mammalian gastrointestinal tract. Significant gaps in knowledge about which gastrointestinal communities and host factors promote plasmid transfer remain. Here, we used Salmonella enterica serovar Kentucky strain CVM29188 carrying plasmid pCVM29188_146 (harboring streptomycin and tetracycline resistance genes) to assess plasmid transfer to Escherichia coli under *in vitro* conditions and in various mouse strains with a conventional or defined microbiota. As an initial test, the transfer of pCVM29188_146 to the E. coli strains was confirmed *in vitro*. Colonization resistance and, therefore, a lack of plasmid transfer were found in wild-type mice harboring a conventional microbiota. Thus, mice harboring the altered Schaedler flora (ASF), or ASF mice, were used to probe for host factors in the context of a defined microbiota. To assess the influence of inflammation on plasmid transfer, we compared interleukin-10 gene-deficient 129S6/SvEv ASF mice (proinflammatory environment) to wild-type 129S6/SvEv ASF mice and found no difference in transconjugant yields. In contrast, the mouse strain influenced plasmid transfer, as C3H/HeN ASF mice had significantly lower levels of transconjugants than 129S6/SvEv ASF mice. Although gastrointestinal members were identical between the ASF mouse strains, a few differences from C3H/HeN ASF mice were detected, with C3H/HeN ASF mice having significantly lower abundances of ASF members 356 (*Clostridium* sp.), 492 (Eubacterium plexicaudatum), and 502 (*Clostridium* sp.) than 129S6/SvEv ASF mice. Overall, we demonstrate that microbiota complexity and mouse genetic background influence *in vivo* plasmid transfer.

**IMPORTANCE** Antibiotic resistance is a threat to public health. Many clinically relevant antibiotic resistance genes are carried on plasmids that can be transferred to other bacterial members in the gastrointestinal tract. The current study used a murine model to study the transfer of a large antibiotic resistance plasmid from a foodborne *Salmonella* strain to a gut commensal E. coli strain in the gastrointestinal tract. We found that different mouse genetic backgrounds and a different diversity of microbial communities influenced the level of Escherichia coli that acquired the plasmid in the gastrointestinal tract. This study suggests that the complexity of the microbial community and host genetics influence plasmid transfer from donor to recipient bacteria.

## Abstract (precis)

Antibiotic resistance is a threat to public health. Many clinically relevant antibiotic resistance genes are carried on plasmids that can be transferred to other bacterial members in the gastrointestinal tract. The current study used a murine model to study the transfer of a large antibiotic resistance plasmid from a foodborne *Salmonella* strain to a gut commensal E. coli strain in the gastrointestinal tract. We found that different mouse genetic backgrounds and a different diversity of microbial communities influenced the level of Escherichia coli that acquired the plasmid in the gastrointestinal tract. This study suggests that the complexity of the microbial community and host genetics influence plasmid transfer from donor to recipient bacteria.

## INTRODUCTION

The spread of antibiotic resistance (AR) and the emergence of multidrug resistance (MDR) reduce the efficacy of treatment of infectious diseases, threatening animal and public health. In the United States alone, an estimated 2 million people are affected by an antibiotic-resistant infection each year (1). Therefore, there is a growing need to address the problem of AR. The primary means for the spread of AR is by horizontal gene transfer (HGT), which has greatly contributed to the coevolution of bacteria (2). Additionally, conjugative plasmids are the principal vectors for HGT of virulence and AR genes, leading to the rapid rise of AR in bacterial pathogens (3, 4). Thus, systems to study AR gene reservoirs are needed to uncover the factors that influence plasmid transfer.

Plasmid transfer has been studied extensively in low-complexity *in vitro* systems (5), and a number of reports have demonstrated the occurrence of plasmid transfer in the mammalian intestine (6). Comparatively few studies have evaluated factors affecting plasmid transfer in a complex environment like the gastrointestinal tract, a reservoir for AR genes (7, 8), where plasmid transfer could be regulated differently from the manner in which it is regulated *in vitro* (9, 10). Mice serve as a model system to explore plasmid transfer in the gastrointestinal tract (11). A few factors, such as antibiotic treatment (12) and inflammation (2), that foster AR-associated plasmid transfer in the gastrointestinal tract have been uncovered using mouse models, but many others have yet to be assessed.

The mammalian gastrointestinal tract harbors a vast number of microorganisms that could carry and transfer AR genes. *Enterobacteriaceae* (*Enterobacterales* ord. nov.) members, such as Escherichia coli and *Salmonella*, can cause clinical enteric infections in humans and often carry plasmids harboring AR genes (AR plasmids) (13). In E. coli, it is estimated that an average of 17% of its genome was acquired from other bacterial species through HGT (14). Nonpathogenic strains represent a large portion of the microbial community in healthy individuals (15, 16), and nonpathogenic E. coli strains have been shown to be a possible hub for the transfer of AR genes to other bacterial strains (17, 18). To study plasmid transmission between *Enterobacteriaceae* strains, previous work has used streptomycin treatment to decrease the numbers of facultative anaerobic bacteria and allow inoculated strains to establish gastrointestinal colonization in mice (19–21). Limitations of this approach include the requirement for streptomycin treatment, often given in drinking water, which can have confounding effects, such as increased intestinal inflammation (22). Recently, we and others have used mice with a defined intestinal microbiota to explore pathogenic and nonpathogenic *Enterobacteriaceae* colonization and diseases (2, 23–25). These mice harbor defined communities that can be easily probed by quantitative PCR (qPCR) methods to quantify the abundance of each member (26).

To investigate whether mouse genetics and inflammation influence plasmid transfer, this study assessed the extent to which a large AR plasmid transferred from a foodborne MDR *Salmonella* strain to nonpathogenic E. coli strains (e.g., the K-12 strain and gut commensal bacteria) under laboratory conditions and in the mouse intestine. We assessed whether the transconjugant yield was affected by the recipient E. coli strain under *in vitro* conditions. We performed an *in vivo* experiment to determine whether the same plasmid transfer could occur in mice colonized with a complex, conventional microbiota (CONV mice). Due to colonization resistance in CONV mice, we reasoned that mice harboring a defined microbiota (mice harboring the altered Schaedler flora [ASF], or ASF mice) would be a better system to explore plasmid transfer because of the ability of ASF mice to be stably colonized by enteric bacteria (23, 24). To assess the influence of inflammation on plasmid transfer, we compared interleukin-10 (IL-10) gene-deficient (*IL-10^−/−^*) 129S6/SvEv ASF mice (proinflammatory environment) to wild-type 129S6/SvEv ASF mice. We also compared how mouse genetic background (C3H/HeN ASF mice versus 129S6/SvEv ASF mice) affected plasmid transfer. ASF members in C3H/HeN ASF mice and 129S6/SvEv ASF mice were quantified after E. coli recipient inoculation, after *Salmonella* donor inoculation, and after exposure to a chemical irritant, dextran sodium sulfate (DSS). Overall, we discovered that plasmid transfer is influenced by mouse genetic background and the complexity of the gut microbial community.

## RESULTS

### The recipient strain impacts the transconjugant yield *in vitro*.

To assess whether the 146-kb plasmid pCVM29188_146 (harboring streptomycin and tetracycline resistance genes) would transfer from Salmonella enterica serovar Kentucky CVM29188 to nonpathogenic E. coli recipient strains, standard *in vitro* mating cultures were used. Samples were serially diluted and plated on MacConkey agar supplemented with nalidixic acid and tetracycline to select for transconjugants with chromosomal (for nalidixic acid resistance) and plasmidic (for tetracycline resistance) AR genes. The transconjugants were further validated by plasmid profiling to contain the conjugally acquired large plasmid pCVM29188_146 (see Fig. S1 in the supplemental material). The mean transconjugant yield obtained when using donor strain MGN026 was 5.4 log_10_ CFU/ml, which was significantly greater (*P = *0.005) than the yield of 3.8 log_10_ CFU/ml obtained when using strain HS-4.

10.1128/mSphere.00847-19.1 FIG S1 Plasmid profile of transconjugants. Plasmid profiles of strains in a 0.5% agarose gel stained with ethidium bromide. R, recipient E. coli HS-4; 1 to 8, transconjugants containing pCVM29188_146; L, three plasmids (of 147 kb, 163 kb, and 35.85 kb) of strain 39R681 used as a ladder (52). Na^r^, nalidixic acid resistant; Tet^r^, tetracycline resistant; Strp^r^, streptomycin resistant. Download FIG S1, TIF file, 0.5 MB. Copyright © 2020 Ott et al. 2020 Ott et al. This content is distributed under the terms of the Creative Commons Attribution 4.0 International license.

### CONV mice display colonization resistance to *Salmonella* strain CVM29188.

**FIG 1:**
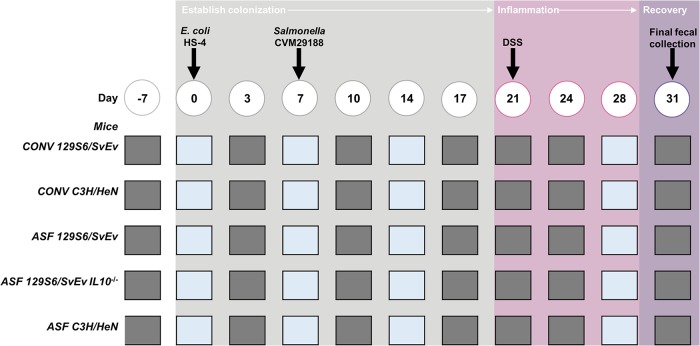
Experimental setup. Mice harbored a gastrointestinal community consisting of the altered Schaedler flora (ASF) or a conventional microbiota (CONV). One week before the inoculation of 10^8^ CFU of Escherichia coli strain HS-4 on day 0, fecal samples were collected to confirm the E. coli- and *Salmonella*-negative status of the mice. On day 7, the mice were inoculated with 10^8^ CFU (unless otherwise indicated) of *Salmonella* strain CVM29188. On day 21, dextran sodium sulfate (DSS) was added to the drinking water at a final concentration of 2% and remained there until day 28 for all mice except *IL-10*^−/−^ 129S6/SvEv ASF mice, which did not receive DSS. Fecal samples were collected only to quantify the bacterial strains by agar plating (gray squares) or for quantifying both bacterial strains by agar plating and microbial members of the gastrointestinal tract by qPCR (blue squares).

To determine the effect of the donor concentration, 129S6/SvEv ASF mice were orally inoculated with HS-4, and this recipient strain was allowed to establish colonization for 7 days before oral inoculation with the plasmid donor, *S.* Kentucky strain CVM29188 (Fig. 1). Selection of HS-4 for use as a recipient strain for *in vivo* experiments was based on its origin as a human gut commensal strain and because it was previously shown to colonize the streptomycin-treated murine intestine (19). Before inoculation, fecal samples were collected from all mice, and their E. coli- and *Salmonella*-negative status was confirmed. To quantify colonization, fecal samples were collected on days 0, 3, 7, 10, 14, 17, 21, 24, 28, and 31 after HS-4 inoculation. In fecal samples from 129S6/SvEv mice with a conventional microbiota (129S6/SvEv CONV mice), the concentrations of HS-4 ranged from 10^4^ to 10^6^ CFU/g and the concentrations of CVM29188 ranged from not detected to 10^5^ CFU/g, but no transconjugants were detected by our plating method (Fig. S2A). Similarly, in fecal samples from C3H/HeN CONV mice, the concentrations of HS-4 ranged from 10^3^ to 10^6^ CFU/g, but neither CVM29188 nor transconjugants were detected (Fig. S2B). For the 129S6/SvEv CONV mice that were positive for a given bacterial strain, HS-4 was detected in 4 of 4 mice, but CVM29188 was detected in only 1 or 2 of 4 mice, depending on the sampling day (Fig. S3). For C3H/HeN CONV mice, HS-4 was detected in 4 of 4 mice and CVM29188 was not detected. For both 129S6/SvEv CONV and C3H/HeN ASF mice, no transconjugants were detected by our plating method in feces at different time points (Fig. S2) or in tissues and intestinal contents after necropsy (Table S1). Negative samples showed no growth following an enrichment step (Table S1). These data demonstrate that these CONV mice, regardless of strain, were insufficiently colonized by CVM29188 and, thus, poor models for studying plasmid transfer among *Enterobacteriaceae* bacteria *in vivo*.

10.1128/mSphere.00847-19.2 FIG S2 Ability of Escherichia coli and *Salmonella* to colonize mice harboring a conventional (CONV) microbiota. Direct plate counts from fecal pellets of 129S6/SvEv CONV mice (*n* = 4) (A) and C3H/HeN CONV mice (*n* = 4) (B). Each symbol represents an individual animal, solid black lines represent the mean for strain HS-4, and solid gray lines represent the mean for strain CVM29188. The results for recipient HS-4 are shown as blue circles, those for the donor are shown CVM29188 as purple squares, transconjugant yields from 129S6/SvEv CONV mice are shown as red diamonds, and transconjugant yields from C3H/HeN CONV mice are shown as red triangles. The horizontal dashed line is the limit of quantification (LOQ). Samples that tested negative were assigned a value halfway between 0 and the LOQ, and undiluted samples with greater than 0 but less than 20 colonies were assigned a value of the LOQ. Download FIG S2, TIF file, 0.3 MB. Copyright © 2020 Ott et al. 2020 Ott et al. This content is distributed under the terms of the Creative Commons Attribution 4.0 International license.

10.1128/mSphere.00847-19.3 FIG S3 Inflammation does not affect transconjugant yield in 129S6/SvEv mice. Direct plate counts from fecal pellets from wild-type 129S6/SvEv mice harboring the altered Schaedler flora (ASF) treated with 2% DSS on day 21 (A), IL-10 gene-deficient (*IL-10*^−/−^) 129S6/SvEv ASF mice (B), and an overlay of the transconjugant yields from DSS-treated (diamond) and IL-10 gene-deficient (*IL-10*^−/−^) 129S6/SvEv ASF mice (triangle) (C) are shown. Recipient Escherichia coli HS-4 is represented by blue symbols, donor *Salmonella* Kentucky CVM29188 is represented by purple symbols, transconjugant yields from wild-type 129S6/SvEv ASF mice are represented as red diamonds, and transconjugant yields from *IL-10*^−/−^ 129S6/SvEv ASF mice are represented as red triangles. Each symbol represents the mean, and error bars represent standard deviations. The horizontal dashed line is the limit of quantification (LOQ). Samples that tested negative were assigned a value halfway between 0 and the LOQ, and undiluted samples with greater than 0 but less than 20 colonies were assigned a value of the LOQ. Significant differences, as indicated by an asterisk (*P < *0.05), were determined by a *t* test. Download FIG S3, TIF file, 0.2 MB. Copyright © 2020 Ott et al. 2020 Ott et al. This content is distributed under the terms of the Creative Commons Attribution 4.0 International license.

10.1128/mSphere.00847-19.6 TABLE S1 Log_10_ number of CFU per gram of tissue or gastrointestinal content of strains at necropsy in tissues and gastrointestinal contents of ASF and conventional mice. Download Table S1, XLSX file, 2.6 MB. Copyright © 2020 Ott et al. 2020 Ott et al. This content is distributed under the terms of the Creative Commons Attribution 4.0 International license.

### The donor concentration impacts the transconjugant yield only on initial sampling of 129S6/SvEv ASF mice.

**FIG 2:**
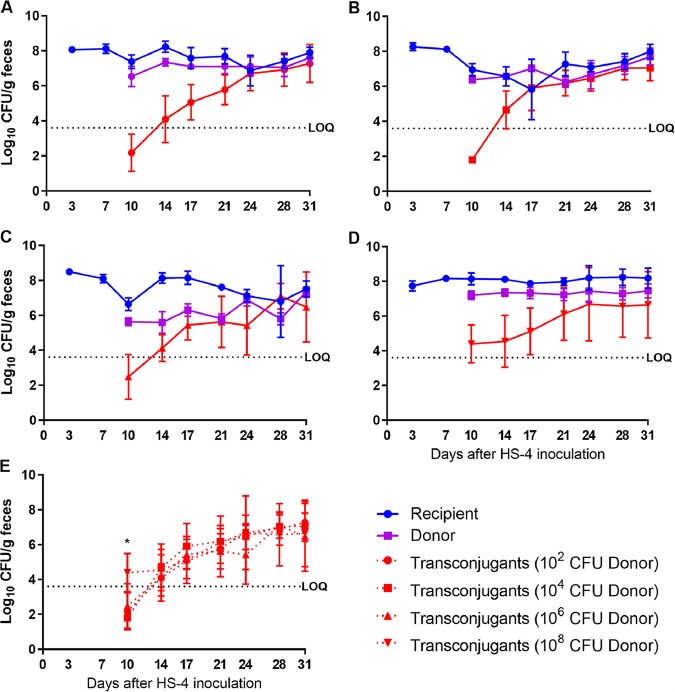
The donor inoculum concentration does not greatly impact the transconjugant yield. Direct plate counts from fecal pellets of 129S6/SvEv mice harboring the altered Schaedler flora inoculated on day 0 with 10^8^ CFU of recipient Escherichia coli HS-4 (blue symbols) and on day 7 with 10^2^ (A), 10^4^ (B), 10^6^ (C), or 10^8^ (D) CFU of donor *Salmonella* Kentucky CVM29188 (purple symbols) were determined. (E) Transconjugants from each donor concentration are overlaid (red symbols). Each symbol represents the mean (8 mice/group), and error bars represent standard deviations. Significant differences (*P < *0.05), as indicated by an asterisk, were determined by an ANOVA, followed by Tukey’s test for multiple means comparison. The horizontal dashed line is the limit of quantification (LOQ). Samples that tested negative were assigned a value halfway between 0 and the LOQ, and undiluted samples with greater than 0 but less than 20 colonies were assigned a value of the LOQ.

We previously found that diarrheagenic and nonpathogenic strains of E. coli colonized the gastrointestinal tract of ASF mice after a single oral inoculation and without the use of antibiotics (23, 24). Thus, we used ASF mice to explore the factors that influence conjugation for the subsequent experiments. To determine the effect of donor concentration, 129S6/SvEv ASF mice were orally inoculated with HS-4, and this recipient strain was allowed to establish colonization for 7 days. On day 7, mice were inoculated with 10^2^, 10^4^, 10^6^, or 10^8^ CFU of CVM29188. At 10 days after HS-4 inoculation, all mice demonstrated fecal concentrations of HS-4 of 10^7^ CFU/g and of CVM29188 of ∼10^6^ CFU/g, and on day 17, all groups established CVM29188 colonization with concentrations of ∼10^7^ CFU/g. On day 10, transconjugants were detected in 1 of 8 mice inoculated with 10^2^ CFU of CVM29188, 0 of 8 mice inoculated with 10^4^ CFU of CVM29188, 2 of 8 mice inoculated with 10^6^ CFU of CVM29188, and 8 of 8 mice inoculated with 10^8^ CFU of CVM29188. The only significant differences in transconjugant yields were found on day 10, with the group inoculated with 10^8^ CFU of CVM29188 having significantly higher levels than all other groups (10^8^ CFU versus 10^2^ CFU, *P = *0.002; 10^8^ CFU versus 10^4^ CFU, *P = *0.0003; 10^8^ CFU versus 10^6^ CFU, *P = *0.009) (Fig. 2). By day 14, transconjugants were detected in all but one mouse (in the group inoculated with 10^4^ CFU of CVM29188). Transconjugants reached a terminal concentration of 10^6^ CFU/g of feces (Fig. 2E). Data obtained from 129S6/SvEv ASF mice inoculated with 10^8^ CFU of CVM29188 were used for subsequent comparisons that assessed the impact of inflammation and mouse genetic background.

### *IL-10^−/−^* genetic background and DSS treatment do not promote a greater transconjugant yield.

Following the same inoculation methods with strains HS-4 and CVM29188 described above, *in vivo* conjugation assays were performed with wild-type 129S6/SvEv mouse and mouse inflammation models, including ASF mice treated with DSS on days 21 through 28 and *IL-10^−/−^* 129S6/SvEv ASF mice. The transconjugant yield was significantly higher (*P = *0.046) in wild-type ASF mice than in *IL-10^−/−^* ASF mice on day 10 (Fig. S3). No other significant differences were detected.

**FIG 3:**
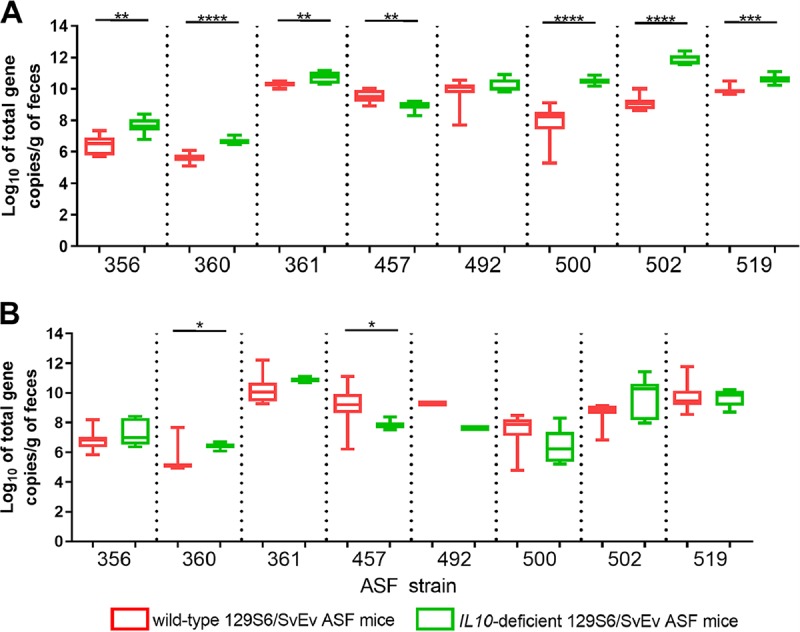
Quantification of altered Schaedler flora (ASF) members in wild-type 129S6/SvEv ASF mice and *IL-10^−/−^* 129S6/SvEv ASF mice. DNA was extracted from fecal samples from wild-type 129S6/SvEv ASF mice (red) and *IL-10^−/−^* 129S6/SvEv ASF mice (green) collected 14 (A) and 28 (B) days after recipient Escherichia coli HS-4 inoculation. Wild-type 129S6/SvEv ASF mice were treated with 2% dextran sodium sulfate (DSS) in their drinking water on day 21 for 7 days, whereas*IL-10^−/−^* mice were not given DSS. Data are represented as box-and-whisker plots of the qPCR values for 7 or 8 mice per group. A *t* test was used to compare the log_10_ total gene copy number per gram of feces between wild-type and *IL-10^−/−^* mice. *P* values of <0.05 were considered significant. *, *P < *0.05; **, *P < *0.01; ***, *P < *0.001; ****, *P < *0.0001.

To assess whether DSS treatment and IL-10 deficiency influenced ASF strain abundances, qPCR was performed on DNA extracted from fecal samples collected on days 14 and 28. Prior to DSS treatment of wild-type 129S6/SvEv mice, there were significant differences in the log_10_ gene copy numbers per gram of feces for all ASF strains except ASF strain 492 (Fig. 3A) when comparing samples collected from the wild-type versus *IL-10^−/−^* 129S6/SvEv ASF mice. Following the initiation of acute gastrointestinal inflammation by a 7-day oral treatment with 2% DSS in the drinking water, there were no longer significant differences between DSS-treated wild-type 129S6/SvEv ASF mice and *IL-10*^−/−^ 129S6/SvEv ASF mice for ASF strain 356, 361, 492, 500, 502, or 519 (Fig. 3B). However, on day 28, ASF strain 360 levels remained significantly higher (*P = *0.02) in *IL-10*^−/−^ ASF mice than in wild-type ASF mice, and ASF strain 457 levels remained significantly higher (*P = *0.04) in wild-type ASF mice than in *IL-10*^−/−^ ASF mice.

### Mouse genetic background affects plasmid transfer.

**FIG 4:**
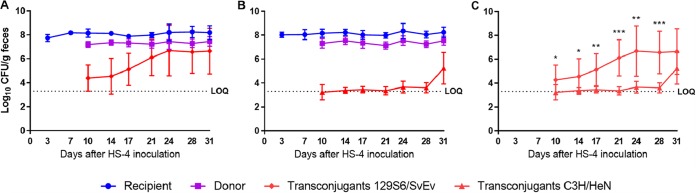
Mouse genetic background affects transconjugant yield. Direct plate counts from fecal pellets from 129S6/SvEv ASF mice (A) and C3H/HeN ASF mice (B) and an overlay of the transconjugant yields from 129S6/SvEv ASF mice (red diamonds) and C3H/HeN ASF mice (red triangles) (C) are shown. Mice were inoculated with recipient Escherichia coli HS-4 (blue symbols) on day 0 and donor *Salmonella* Kentucky CVM29188 (purple symbols) on day 7. Data are representative of those from two individual experiments. Each symbol represents the mean (8 mice/group), and error bars represent standard deviations. The horizontal dashed line is the limit of quantification (LOQ). Samples that tested negative were assigned a value halfway between 0 and the LOQ, and undiluted samples with greater than 0 but less than 20 colonies were assigned a value of the LOQ. Significant differences (*P < *0.05) were determined by a *t* test. *, *P < *0.05; **, *P < *0.01; ***, *P < *0.001.

The impact of the mouse strain was examined by conducting *in vivo* conjugation assays with 129S6/SvEv and C3H/HeN ASF mice. Using the same inoculation methods used for the conjugation experiments described above, both mouse strains demonstrated fecal concentrations of HS-4 and CVM29188 of 10^8^ CFU/g and 10^7^ CFU/g, respectively. On days 10 through 28, 129S6/SvEv mice demonstrated significantly increased (*P < *0.05) levels of transconjugants in fecal samples compared with those in C3H/HeN ASF mice (Fig. 4C). On day 10, 129S6/SvEv and C3H/HeN ASF mice presented transconjugant concentrations of 10^4^ and 10^3^ CFU/g of feces, respectively. In 129S6/SvEv ASF mice, the transconjugant yield steadily increased to a maximal concentration of 10^6^ CFU/g of feces on day 24 and was maintained at that level for the remainder of the study. In contrast, in C3H/HeN mice, transconjugant yields remained at about 10^3^ CFU/g of feces, with a slight increase over time to a maximal concentration of 10^5^ CFU/g of feces on day 31.

**FIG 5:**
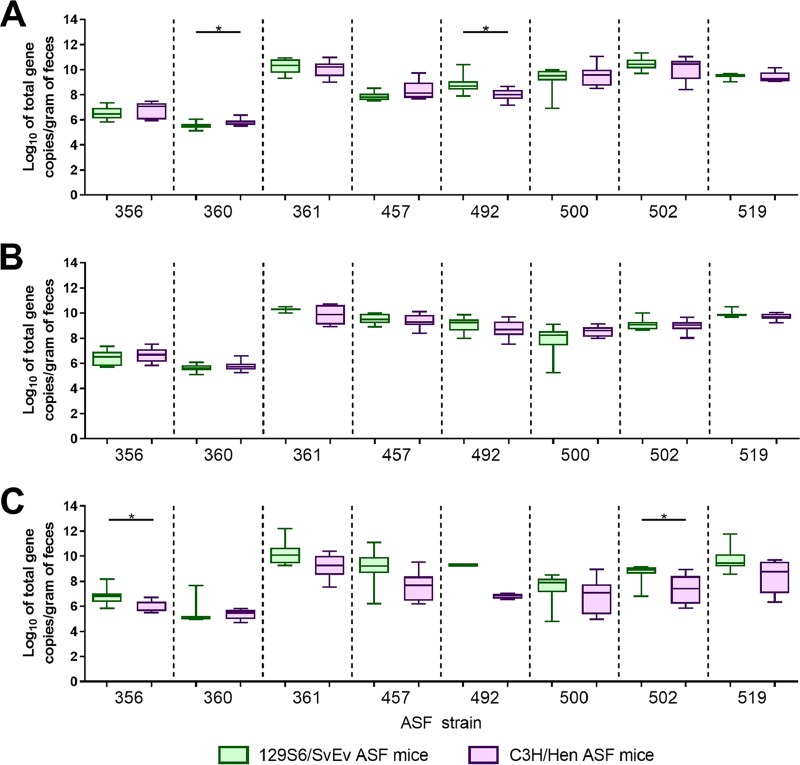
Quantification of ASF strains in 129S6/SvEv and C3H/HeN mice harboring the altered Schaedler flora (ASF). At 7 (A), 14 (B), and 28 (C) days after HS-4 inoculation, DNA was extracted from fecal samples from 129S6/SvEv (green) and C3H/HeN ASF (purple) mice (*n* = 8/group). Data are representative of those from two individual experiments and are displayed as box-and-whisker plots of qPCR values. A *t* test was used to compare the log_10_ total gene copy number per gram of feces between 129S6/SvEv and C3H/HeN ASF mice. *P* values of <0.05 were considered significant and are indicated by an asterisk.

Although 129S6/SvEv ASF mice and C3H/HeN ASF mice are colonized with identical microbiota members, differences in the abundances of these members could facilitate changes in interactions between HS-4 and CVM29188. To assess whether ASF strain abundances were different between 129S6/SvE and C3H/HeN ASF mice following colonization with HS-4 and CVM29188, qPCR was performed on DNA extracted from fecal samples collected on days 7, 14, and 28. On day 7, the level of ASF strain 360 (Lactobacillus murinus) was significantly higher (*P = *0.04) in C3H/HeN mice than in 129S6/SvEv mice, whereas the level of ASF strain 492 (Eubacterium plexicaudatum) was significantly higher (*P = *0.02) in 129S6/SvEv mice than in C3H/HeN mice (Fig. 5A). On day 14, no significant differences were detected (Fig. 5B). On day 28, the levels of ASF strain 356 (a *Clostridium* sp.) and ASF strain 502 (a *Clostridium* sp.) were significantly higher (*P = *0.03 for both strains) in 129S6/SvEv mice than in C3H/HeN mice (Fig. 5C).

In order to compare ASF strain abundances over time in the mice used in the conjugation experiments, the level of each ASF strain was compared within each mouse background. In general, ASF strain abundances decreased over time regardless of the mouse strain used (Fig. S4). Specifically, for 129S6/SvEv ASF mice, there were no significant changes in the abundances of ASF strains 356 (a *Clostridium* sp.), 360 (Lactobacillus intestinalis), 361 (Lactobacillus murinus), 492 (Eubacterium plexicaudatum), and 519 (Parabacteroides goldsteinii) over time. In contrast in 129S6/SvEv ASF mice, ASF strain 457 (Mucispirillum schaedleri) significantly increased from day 7 to days 14 and 28, whereas ASF strain 500 (a *Pseudoflavonifractor* sp.) significantly decreased in abundance from day 7 to day 28 and ASF strain 502 (a *Clostridium* sp.) significantly decreased in abundance from day 7 to days 14 and 28. For C3H/HeN ASF mice, we identified several significant differences that were not found in 129S6/SvEv mice. Consistently, all 8 ASF taxa had significantly decreased levels from days 0 to 28 in C3H/HeN ASF mice.

10.1128/mSphere.00847-19.4 FIG S4 Changes in altered Schaedler flora (ASF) member abundances over time within the mouse strains. On day 0, a fecal sample was collected before inoculation with recipient Escherichia coli strain HS-4. DNA was extracted from fecal samples collected on days 0, 7, 14, and 28 from 129S6/SvEv ASF mice, IL-10 gene-deficient (*IL-10*^−/−^) 129S6/SvEv ASF mice, 129S6/SvEv mice with a conventional microbiota (CONV mice), C3H/HeN CONV mice, and C3H/HeN ASF mice (*n* = 8/group for ASF mice, *n* = 4/group for CONV mice). All mice were inoculated with 10^8^ CFU of donor strain *Salmonella* CVM29188 on day 7, except for 129S6/SvEv ASF mice, which were inoculated with 10^2^, 10^4^, or 10^6^ CFU of CVM29188, as indicated. Data are represented as box-and-whisker plots of qPCR values. An ANOVA followed by Tukey’s test for multiple comparisons was used to compare the log_10_ total gene copy number per gram of feces. *P* values of <0.05 were considered significant. *, *P < *0.05; **, *P < *0.01; ***, *P < *0.001; ****, *P < *0.0001; NDA, no data available; ND, not detected. Download FIG S4, TIF file, 0.7 MB. Copyright © 2020 Ott et al. 2020 Ott et al. This content is distributed under the terms of the Creative Commons Attribution 4.0 International license.

## DISCUSSION

Conjugative plasmids have been identified to be key vectors for spreading DNA, which can confer AR and MDR (27–29). The evaluation of plasmid transfer under *in vitro* conditions serves as a low-complexity test to determine the compatibility of transfer to recipient strains. Previously, Fricke and colleagues (30) demonstrated the transfer of a 146-kb plasmid harboring tetracycline and streptomycin resistance genes (pVCM29188_146) to *S*. Kentucky CVM29188, *S*. Newport SL317, and E. coli DH10B *in vitro* but not *in vivo*. Here, we showed that the recipient strain influenced the transconjugant yield *in vitro*, which confirms the previous findings (30). The methods used in this study match those used in traditional *in vitro* studies; however, future *in vitro* studies could better model the gut environment by accounting for various factors, such as pH, salinity, and oxygen and carbon dioxide concentrations. Although the use of recipient strain MGN026 resulted in transconjugant yields higher than those obtained by the use of HS-4 *in vitro*, transconjugants were still found using HS-4. Instead of the DH5α derivative strain MGN026, HS-4 was used as a recipient strain *in vivo* because it is a human gut commensal E. coli strain and it has previously been shown to colonize the mouse intestine (19).

CONV mice provide a complex gastrointestinal environment to assess microbe-microbe and host-microbe interactions, as *in vitro* conditions do not accurately reflect plasmid transfer under *in vivo* conditions (31, 32). Although detectable levels of HS-4 were found in both 129S6/SvEv and C3H/HeN CONV mice, neither consistent nor persistent colonization with CVM29188 could be achieved and no transconjugants were detected. The choice of animal model should be selected based on the questions to be studied. Previous studies have used CONV (33, 34), antimicrobial-treated (32), defined microbiota (2, 35), and germfree (35, 36) mice to assess plasmid transfer in the gastrointestinal tract of mice. As observed previously and in the current study, CONV animals are often resistant to colonization by orally inoculated bacterial strains, making these animals unreliable when assessing plasmid transfer without additional manipulation (37). Treatment with antimicrobials often allows for colonization with inoculated strains, but antimicrobials can have adverse effects, such as intestinal irritability, including inflammation (22, 32, 38). To our knowledge, a few studies (2, 35) have used mice with a defined microbiota, and only Stecher et al. (2) used ASF mice to assess plasmid transfer. The advantages of using ASF mice include consistent and prolonged colonization of inoculated strains and the fact that methods have been developed to probe each member of the community (26, 39).

Factors such as antibiotic treatment (12) and inflammation (2) have been shown to influence plasmid transfer. To investigate the role of inflammation in our system, we used *IL-10*^−/−^ mice and DSS-treated wild-type mice, which are known to develop intestinal inflammation with (*IL-10*^−/−^ mice) (40) and without (DSS-treated mice) (41) the presence of enteric organisms. Our data do not fit the understanding that inflammation increases the transconjugant yield, as the genetically predisposed inflammation model did not significantly change the transconjugant yield. A lack of significant differences for transconjugants in our system may be due to the bacterial mating pairs used in the current study and may not reflect the interaction of inflammation with all *Enterobacteriaceae* strains, necessitating further investigation. We used an *S*. Kentucky strain, whereas Stecher et al. used an *S.* Typhimurium strain (2). Additionally, contrary to our methods, previous studies used a T cell-induced inflammation model. This same method of initiating inflammation could be studied to determine the relationship of inflammation and transconjugant yield with our bacterial strains (2). However, we did find that on day 14 (7 days after CVM29188 inoculation), the relative abundance of six of the eight ASF members was significantly elevated in *IL-10*^−/−^ mice compared to that in wild-type 129S6/SvEv mice. Blooms in members of the microbiota occur when bacterial species that are typically found in low densities (less than 10^8^ CFU/ml) increase to densities exceeding 10^8^ CFU/ml (42). Previously, pathogen infection and genetic predisposition (e.g., IL-10 deficiency) altered microbial communities and resulted in *Enterobacteriaceae* blooms (43–45). Here, we had high levels of HS-4 and CVM29188 throughout the study and did not demonstrate a bloom of either strain.

Alternatively, this study demonstrated that there is a significant difference in transconjugant yields according to host genetics. From our data, inbred mouse strains with different genetic backgrounds, 129S6/SvEv versus C3H/HeN, facilitated different levels of transconjugants in the gastrointestinal tract. Transconjugant levels are a key indication of the spread of the AR plasmid. Various factors, such as oxygen conditions, temperature, hydrogen peroxide concentration, sodium chloride concentration, and bile salt concentration, have been found to influence the transfer of S. enterica serovar Infantis plasmid pESI *in vitro* (34). Further investigation is needed to identify the specific host factors that accounted for the differences in transconjugant yields in the current study. In addition, it was demonstrated that the pESI plasmid was transferred to members of the gastrointestinal microbiota (e.g., Lactobacillus reuteri, *Ruminococcaceae*) of C57BL/6 mice during *Salmonella* infection (34). In our study, both conjugative plasmids of *S.* Kentucky, including pCVM29188_146 (IncFIB/IncFIIA) and pCVM29188_101 (IncI), have a narrow host range (46), which limits the conjugal host range of these plasmids to other members of the family *Enterobacteriaceae*, of which no ASF members are included (47); and the plasmid pCVM29188_46 (IncFII) is not conjugative (30). Plasmid gel electrophoresis confirmed the presence of pCVM29188_146 in our transconjugants, but none of these carried the second AR conjugative plasmid, pCVM29188_101 (see Fig. S1 in the supplemental material). Transconjugants with pCVM29188_101 alone could have been missed because our experiments used medium selective for pCVM29188_146 but not for pCVM29188_101. Studies using ASF mice inoculated with strains carrying plasmids with broader host ranges are ongoing. Ongoing studies in our labs are also currently evaluating the role of host factors and ASF members in the acquisition and transfer of plasmids.

Large plasmids have been shown to present a minimal metabolic burden, allowing them to spread and persist in microbial populations (34). However, changes in the microbiota during these interactions have not been studied in detail. In the current study, to evaluate whether introducing bacteria into ASF mice changes the resident microbial members of the gut, we monitored the abundance of each of the ASF member strains over the course of each *in vivo* experiment. The general trend was that ASF members had decreased abundances over time, which is consistent with pathogen colonization (48). A few transient significant differences in the abundances of ASF strains were detected between 129S6/SvEv ASF mice and C3H/HeN ASF mice, but no trend that could account for the differences in transconjugant yield was found. Thus, it appears that differences in the transfer of this particular plasmid between ASF mouse strains are independent of the ASF microbiota, suggesting that host factors are responsible. Overall, new systems to analyze AR reservoirs like the one used in the current study are urgently needed to uncover factors that influence plasmid transfer and to facilitate the discovery of intervention strategies.

### Conclusions.

This study demonstrates that the recipient bacterial strain influences the transconjugant yield under *in vitro* conditions. CONV mice displayed colonization resistance to donor strain *Salmonella* CVM29188, making them unsuitable for continued investigation. However, ASF mice were consistently colonized with both recipient strain E. coli HS-4 and donor strain *Salmonella* CVM29188. The current study did not find an increase in transconjugant yield in the context of intestinal inflammation in *IL-10*^−/−^ ASF mice (genetic predisposition) and DSS-treated ASF mice (chemical predisposition). By testing plasmid transfer in different mouse strains with the same defined microbiota and under the same conditions (environment and diet), our study shows that the host and the complexity of the gut microbiota are driving factors governing transconjugant yields in the gastrointestinal tract. Furthermore, the stability of both donor and recipient bacterial strain colonization of both ASF mouse strains indicates that host differences between the two mouse strains and not colonization levels had a major impact on transconjugant yields. At present, only a model that controls for host microbiota, environment, and diet, such as the ASF mouse model, permits reliable investigation of this host-microbe interaction. Future studies are needed to understand which host factors significantly affect conjugation in the gut.

## MATERIALS AND METHODS

### Ethics statement.

Animal procedures were approved by the Iowa State University Institutional Animal Care and Use Committee (protocol number 9-04-5755-M). Mice were acclimated for at least 2 days before all experiments.

### Bacterial strains.

The bacterial strains used are listed in Table 1. S. enterica serovar Kentucky strain CVM29188 (GenBank accession number ABAK02000001.1) was isolated from a chicken meat sample (30). CVM29188 contains 3 plasmids, 2 of which carry AR genes, including pVCM29188_146 (146 kb; GenBank accession number CP001122), which carries genes for resistance to aminoglycosides and tetracyclines, and pCVM29188_101 (101 kb; GenBank accession CP001121), which carries genes for resistance to cephalosporins and quaternary ammonium compounds. Spontaneous nalidixic acid-resistant (Nal^r^) mutants of nonpathogenic E. coli strains HS-4 Nal^r^ (GenBank accession number CP000802.1) and MGN026 Nal^r^ (for which no GenBank accession number exists), which lack plasmids, were used as recipient strains and are referred to as HS-4 and MGN026 throughout the article.

**TABLE 1:** Escherichia coli and *Salmonella* strains with associated plasmids

Bacterial strain	Species	Role	Plasmid	Plasmid size (kb)	Antibiotic resistance	Reference or source
CVM29188	S. enterica serovar Kentucky	Donor	pCVM29188_146	146	Aminoglycosides, tetracyclines	30
			pCVM29188_101	101	Cephalosporins, quaternary ammonium compounds	
			pCVM29188_46	46	—^*a*^	
DH5α:ASF16S	E. coli	16S rRNA vector	pCR2.1	3.9	Ampicillin	26
HS-4	E. coli (human gut commensal)	Recipient	None	—	—	50
HS-4 Nal^r^	E. coli	Recipient	None	—	Spontaneous nalidixic acid-resistant mutant	This study
MGN026	E. coli (DH5α derivative)	Recipient	None	—	—	51
MGN026 Nal^r^	E. coli	Recipient	None	—	Spontaneous nalidixic acid-resistant mutant	This study

a —, not applicable.

### *In vitro* mating.

The ability of the donor strain to transfer AR plasmids was examined using a liquid culture mating technique. Mating pairs included (i) CVM29188 and HS-4 and (ii) CVM29188 and MGN026. Strains were grown in 3 ml of lysogeny broth (LB) overnight at 37°C with shaking at 200 rpm. On the next day, the cultures were adjusted to an optical density at 600 nm (OD_600_) of ∼1.0, and 1 ml of culture was centrifuged at 10,000 × *g* for 5 min and resuspended in 0.5 ml of LB. The donor and recipient strains were mixed together at a 1:1 ratio and incubated for 24 h at 37°C. Dilutions of the mating mixtures were plated onto MacConkey agar plates containing antibiotics, as follows: 40 μg/ml nalidixic acid (recipient and transconjugants), 10 μg/ml tetracycline (donor and transconjugants), or 40 μg/ml nalidixic acid and 10 μg/ml tetracycline (transconjugants only). Antibiotics were purchased from Fisher Scientific.

### Model of plasmid transfer in mouse intestine.

Mating pair CVM29188 and HS-4 was used to assess conjugation in mice. Ten- to 12-week-old 129S6/SvEv (*n* = 4) and C3H/HeN (*n* = 4) mice with a conventional microbiota were purchased from Taconic Biosciences. Gnotobiotic C3H/HeN ASF mice (∼17 weeks old, *n* = 8), wild-type 129S6/SvEv ASF mice inoculated with 10^8^ CFU of CVM29188 (∼19 weeks old, *n* = 8), wild-type 129S6/SvEv ASF mice inoculated with 10^2^ to 10^6^ CFU of CVM29188 (10 to 14 weeks old, *n* = 8/group), and *IL-10*^−/−^ 129S6/SvEv ASF mice (10 to 12 weeks old, *n* = 7) were originally obtained from Taconic Biosciences. ASF mice were bred and maintained as previously described (24). Approximately equal numbers of female and male mice were used for each group, expect for C3H/HeN CONV mice, where all males were used due to availability by the supplier. Mice were orally gavaged with 10^8^ CFU of spontaneous nalidixic acid-resistant strain HS-4 (Table 1). Recipient strain colonization was monitored by collecting fecal samples on days 3 and 7 and plating them on MacConkey agar plates supplemented with 40 μg/ml of nalidixic acid before inoculation by oral gavage of 10^2^, 10^4^, 10^6^, or 10^8^ CFU of the donor strain CVM29188 on day 7. Following CVM29188 inoculation, fecal samples were collected every 3 to 4 days, and the aforementioned plating scheme was used to quantify the donor, the recipient, and the transconjugants. Mice were euthanized by carbon dioxide inhalation at the end of the study, and tissues (liver and spleen) and intestinal contents (duodenum, jejunum, ileum, cecum, and colon) were collected for bacterial enumeration. Samples showing no growth of either the donor or the recipient were enriched in LB broth for 16 h and plated on selective medium to verify their absence.

### Typing to verify transconjugants.

Plasmid profiling was conducted as described previously, with minor modifications (49). Briefly, E. coli transconjugant isolates were grown in LB to an OD_600_ of ∼0.8. The cultures were then centrifuged and resuspended in 1 ml 0.04 M Tris-acetate, pH 8.0, 2 mM EDTA. Then, 2 ml of lysis buffer (0.05 M Tris, 3% SDS, pH 12.5) was added, and the components were mixed by inversion. The cultures were incubated at 68°C for 60 min. Hot samples were added to 6 ml of phenol-chloroform (1:1), and the components were gently mixed by inversion. The phases were then separated by centrifugation at 10,000 × *g* for 20 min at room temperature. The top aqueous solution was transferred to a new tube, and gel electrophoresis was used to characterize plasmid bands, as previously described (49).

### Fecal sample collection and total DNA extraction.

Fresh fecal samples were collected aseptically every 3 to 4 days (alternating). Fecal pellets were placed into sterile microcentrifuge tubes, homogenized via completion to 1 ml with sterile phosphate-buffered saline, and resuspended with a pipette. Samples were stored at −80°C prior to DNA extraction. Sample DNA was extracted according to previously described methods (26). Following total DNA extraction, the sample concentration was determined using a NanoDrop Lite spectrophotometer (Thermo Fisher Scientiﬁc, Waltham, MA, USA). Total DNA was serially diluted to 10 ng/μl in sterile cell culture-grade water. Following dilution, the samples were stored at −20°C until qPCR analysis.

### ASF standard curve generation.

E. coli DH5α strains harboring the pCR2.1 cloning vector containing the unique 16S rRNA gene sequence of each ASF taxon member were obtained as generous gifts from Amanda Ramer-Tait (University of Nebraska—Lincoln). Each clone was grown overnight in LB supplemented with kanamycin (50 μg/ml) and ampicillin (225 μg/ml) in a shaker incubator set to 225 rpm and 37°C. Following overnight culture, each of the eight strains was used for plasmid DNA extraction using a QIAprep Spin miniprep kit (Qiagen), following the manufacturer’s instructions. The presence of extracted plasmid DNA was validated by gel electrophoresis. Furthermore, the presence of each ASF taxon’s unique 16S rRNA gene sequence was verified by standard PCR amplification using KlenTaq LA DNA polymerase (DNA Polymerase Technology, Inc.). The PCR conditions were as follows: (i) a single cycle of 94°C for 2 min; (ii) 35 cycles of 94°C for 15 s, 60/62°C for 30 s, and 68°C for 45 s; and (iii) one cycle of 68°C for 2 min and a hold at 4°C. The concentrations of plasmid DNA extracts were estimated by use of a NanoDrop Lite spectrophotometer and further quantified by use of a Qubit (version 2.0) fluorometer and the Qubit double-stranded DNA BR assay (Life Technologies). Plasmid stocks were then serially diluted in sterile nuclease-free water at 5-fold dilutions and used for subsequent quantitative PCR analysis. The resulting data were plotted, and a linear regression was applied to calculate the equation of the line and reaction efficiency (see Fig. S5 in the supplemental material).

10.1128/mSphere.00847-19.5 FIG S5 Linear regression analysis of qPCR standard curves for ASF strain 16S rRNA gene copy enumeration. Standard curves were prepared by serially diluting purified vector-free plasmids in sterile nuclease-free water. Plasmid concentrations were analyzed in triplicate with the predicted linear model, with the mean values (squares) being shown. Dotted lines indicate 95% confidence interval bands. *R*^2^ values and the equations for the lines are shown. Linear regressions were used to determine the limit of quantification (LOQ) and the efficiency of each reaction. Download FIG S5, TIF file, 0.3 MB. Copyright © 2020 Ott et al. 2020 Ott et al. This content is distributed under the terms of the Creative Commons Attribution 4.0 International license.

### ASF bacterial strain quantification.

ASF bacterial species abundance was determined using qPCR, as previously described (26), with modifications. Briefly, the 24-μl KlenTaq LA qPCR master mix supplemented with the EvaGreen reporter dye and the carboxy-X-rhodamine (ROX) reference dye (Biotium) and 1-μl total DNA extracts at 10 ng/μl were aliquoted in duplicate into MicroAmp Fast optical 96-well reaction plates capped with MicroAmp optical adhesive film (Applied Biosystems). The samples were analyzed on a StepOnePlus real-time PCR system (Applied Biosystems) following the manufacturer’s instructions. Threshold cycle (*C_T_*) values were determined by the use of Applied Biosystems StepOnePlus software defaults. Original sample concentrations of bacteria were back calculated by referencing the original mass of the collected fecal sample. Formulas for calculating rRNA operon copy number, total genome copy number, and final genome copy number were previously described elsewhere (26).

### Statistical analysis.

Statistical analyses of the data were performed using GraphPad Prism (version 8) software (San Diego, CA). A *t* test was used to compare the transconjugant yield and ASF member abundances between the different mouse genetic backgrounds and between wild-type and *IL-10*^−/−^ mice. An analysis of variance (ANOVA) was used for comparisons with 3 or more groups in the titration experiments and to compare ASF member abundances within a mouse strain over time. *P* values of ≤0.05 were considered significant.
