# Supplementary figures and images for: Mouse Genetic Background Affects Transfer of an Antibiotic Resistance Plasmid in the Gastrointestinal Tract
Source: mSphere. 2020 Jan 29;5(1):e00847-19. doi: 10.1128/mSphere.00847-19 (PMC6992376; doi:10.1128/mSphere.00847-19)

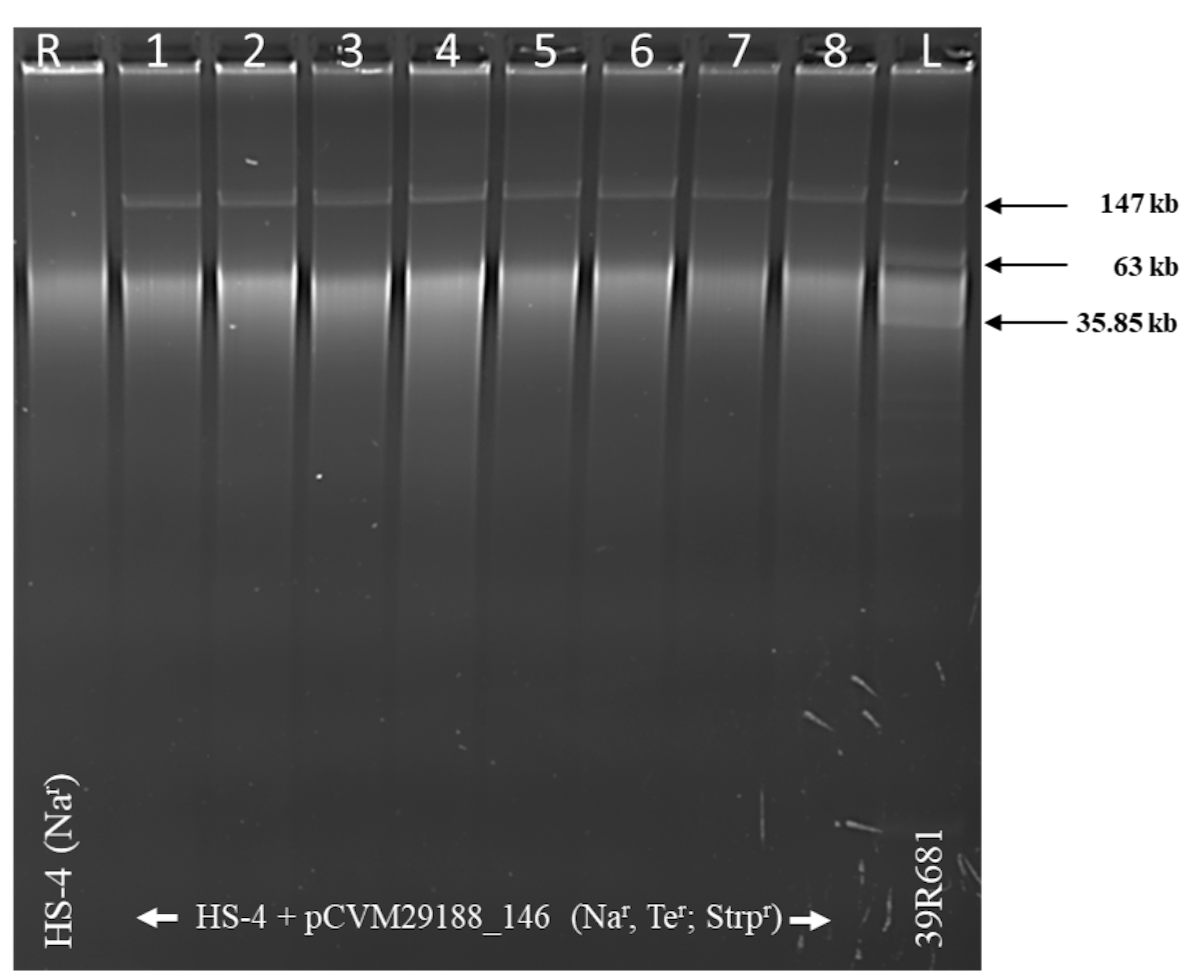

Supplement: FIG S1 [file mSphere.00847-19-sf001.tif]

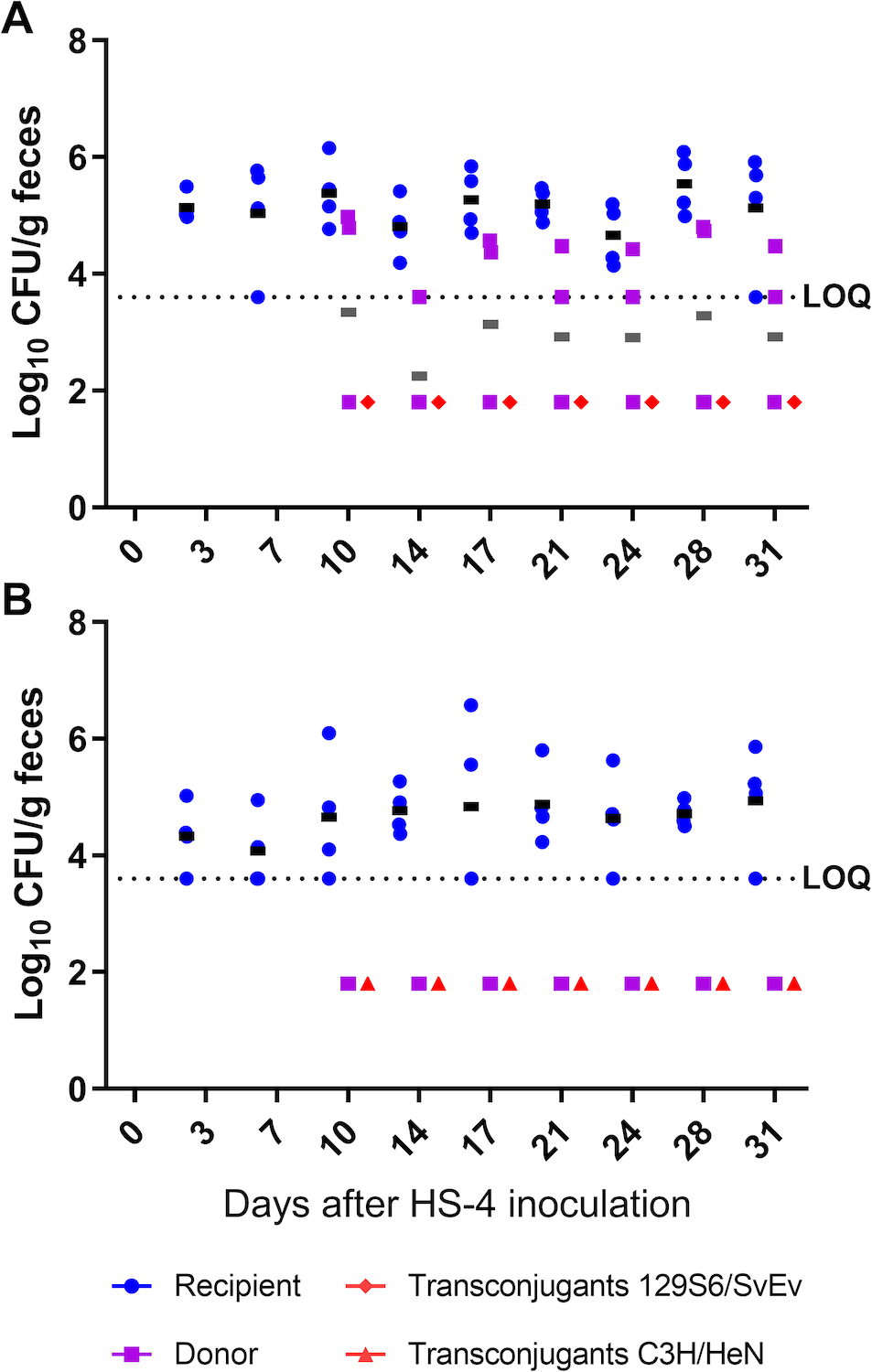

Supplement: FIG S2 [file mSphere.00847-19-sf002.tif]

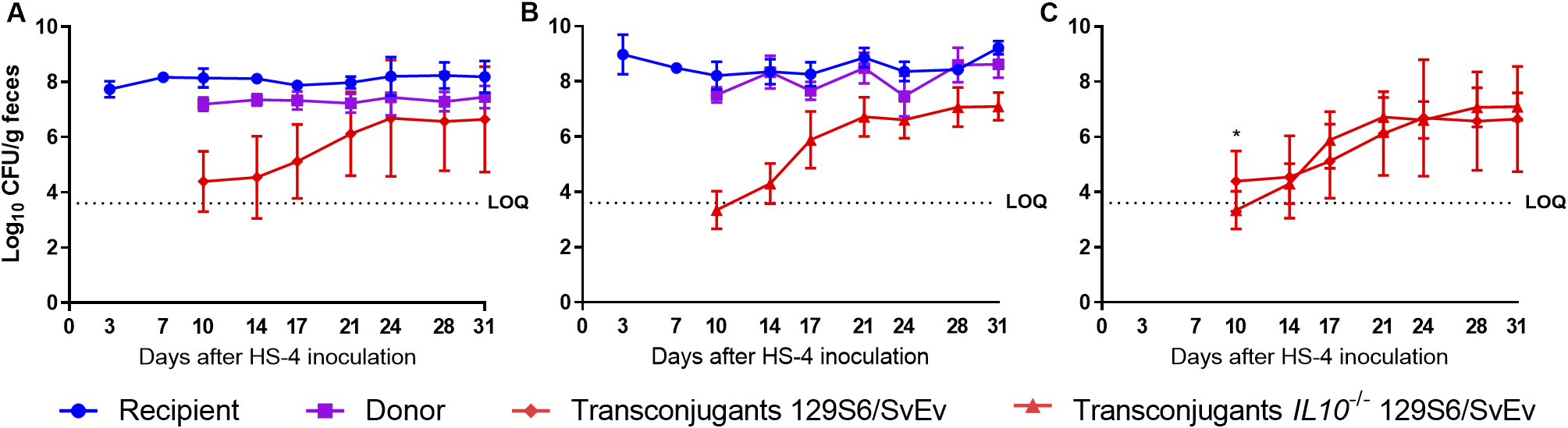

Supplement: FIG S3 [file mSphere.00847-19-sf003.tif]

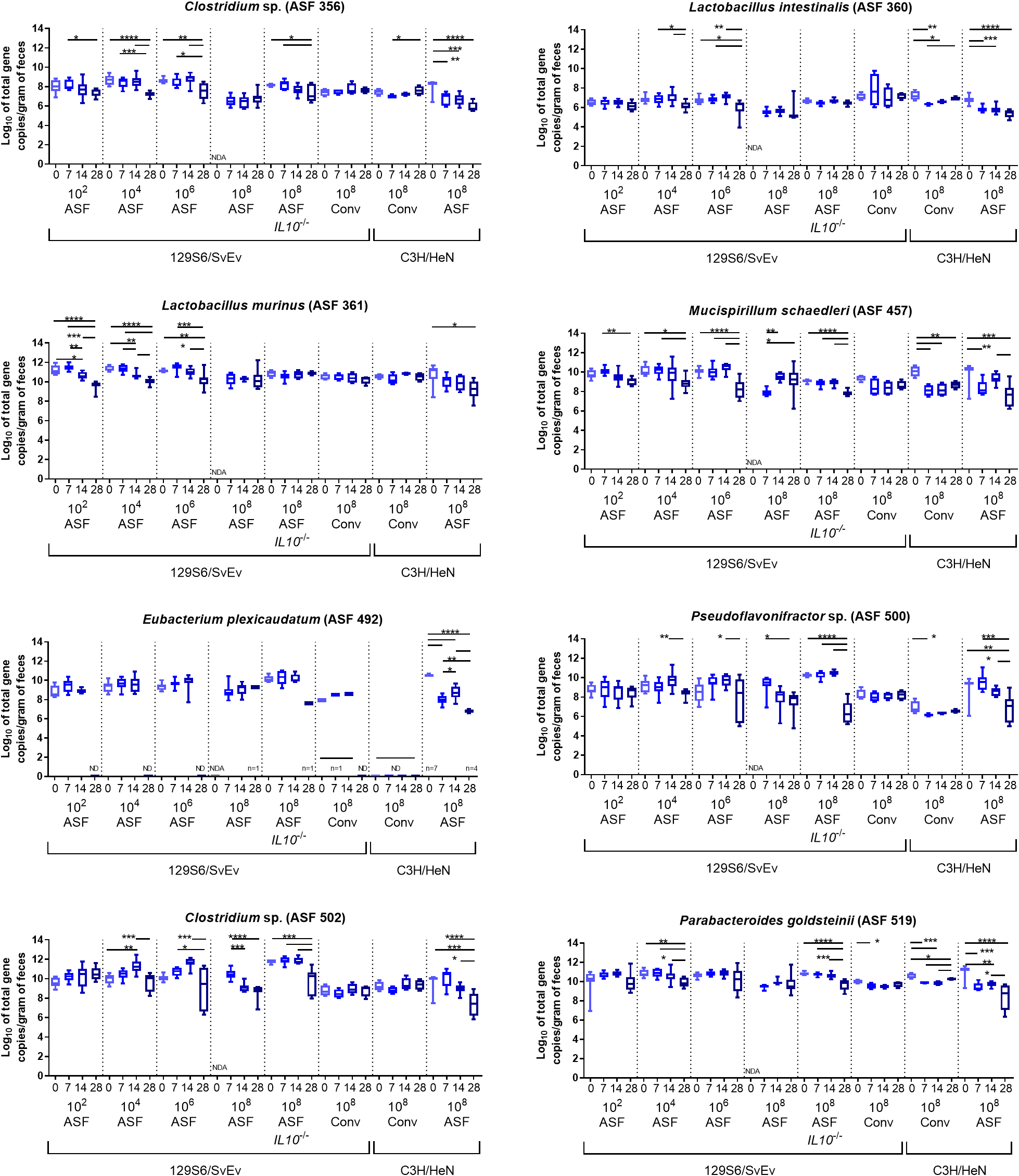

Supplement: FIG S4 [file mSphere.00847-19-sf004.tif]

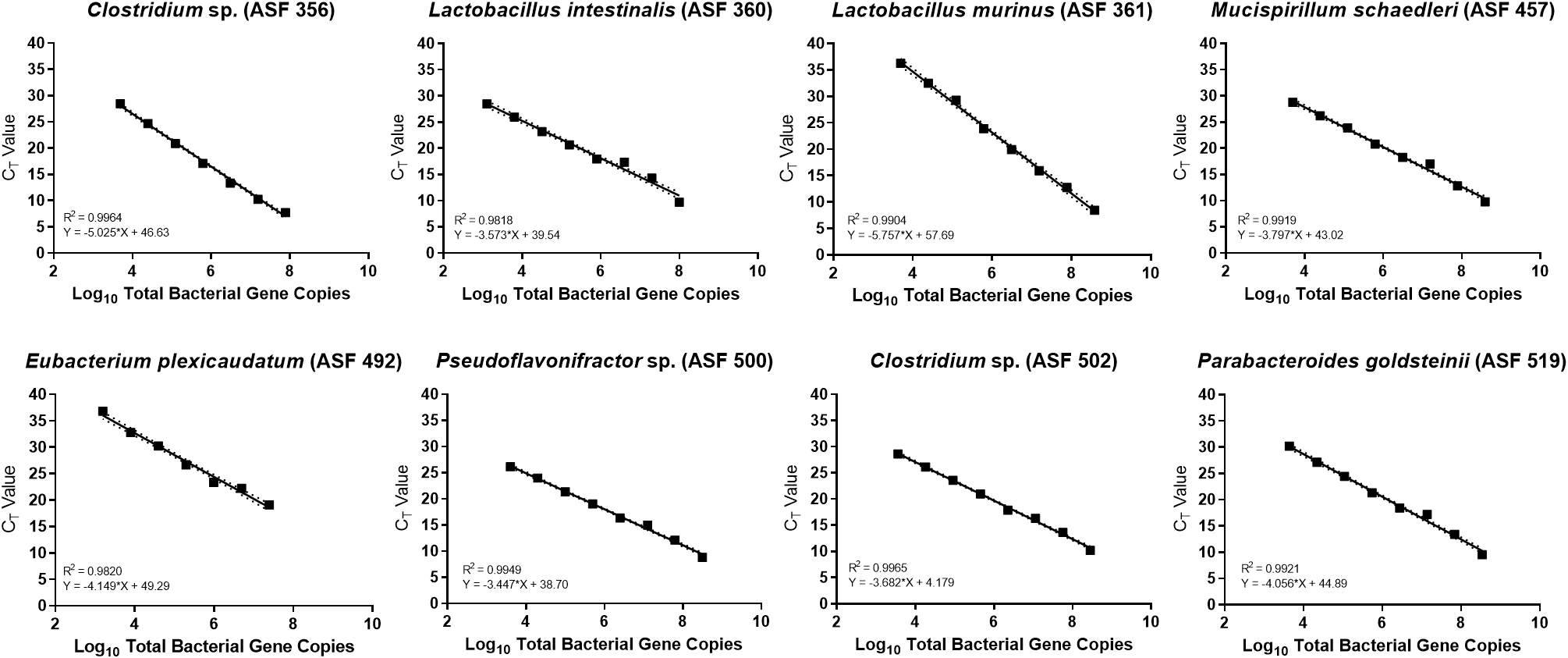

Supplement: FIG S5 [file mSphere.00847-19-sf005.tif]
